# Developing Servant Leadership through Experience and Practice: A Case Study in Service Learning

**DOI:** 10.3390/bs14090801

**Published:** 2024-09-11

**Authors:** Gordon Matthew Robinson, Marshall J. Magnusen

**Affiliations:** 1 Department of Exercise Science and Sport Management, Schreiner University, Kerrville, TX 78028, USA; 2 Department of Educational Leadership, Baylor University, Waco, TX 76706, USA; marshall_magnusen@baylor.edu

**Keywords:** servant leadership, sports management, mentoring, service learning, leadership development, experiential learning, promoting leadership

## Abstract

Servant leadership is characterized by a core altruistic calling and central attributes of genuine caring, humility, and empathy, and in recent years, has become a style of leadership recommended to be addressed in sports management classrooms because of its associated positive outcomes and emphasis on ethical behavior and decision-making. As the relevance and popularity of servant leadership continue to grow, it gives rise to matters of how this approach to leadership can be better studied and taught to current and aspirant sports professionals. Thus, the purpose of this qualitative case study is to analyze a community-based service-learning project as a mode for developing servant leaders as part of a college sports management curriculum. Nineteen upper-level college sports management students participated in this study by serving as mentors in an after-school program for underprivileged elementary school students for 12 h across the course of six weeks. Participants then completed a written reflection upon the conclusion of the service project. Content analysis of these reflections suggests that service learning may be a positive method for developing servant leaders. The article closes with a discussion of findings, proposed future research questions, as well as ideas for future service-learning projects aimed at developing servant leaders.

## 1. Introduction

As business organizations search for forms of leadership that are more pro-social, the focus of leadership studies and practice has shifted from authoritarian styles to less egoistic and more positive forms of leadership centered on fellowship and developing subordinates by meeting their needs and promoting their personal and professional growth [1,2,3]. One style of leadership that aligns with this shift in the modern workplace and that has garnered heightened interest amongst management scholars over the past decade is servant leadership (SL). Servant leadership embodies the core principles of prioritizing others and ethical conduct, which means leaders who embody this style place service to others above personal authority and domination [4]. As the relevance and popularity of SL continue to grow, it gives rise to matters of how this approach to leadership can be better understood and developed in aspirant business professionals such as students [1].

Though numerous academic models of SL have emerged since its inception [5,6,7,8,9,10,11,12], conceptual ambiguity compounded with a proliferation of overlapping descriptive terms within these models can make understanding the exact nature of SL challenging [1]. Indeed, the seeming paradox of the servant as leader and the leader as servant, which represents an inverted leadership hierarchy, can be a challenging concept to grasp when authoritative leadership styles have been dominant for decades in business cultures throughout the world. Thus, because the idea of SL may be confusing at first, especially to inexperienced, aspirant business professionals, focused attention needs to be given to how SL can be better understood and how SL qualities can be developed and put into actual practice in professional settings. Directing scholarly attention to experiential, community-based service learning, for instance, may be an effective mode for developing the next generation of business professionals who can lead with a servant’s heart [1].

Though certain individuals may possess desirable leadership characteristics from birth, such as empathy or being a great listener, many attributes crucial for effective servant leadership can be developed via systematic experiences aimed at promoting the development of specific knowledge and skills [13,14,15,16]. Vertical development through experiential learning, for example, can help future leaders become better equipped with knowledge and how that knowledge can be applied in practice to enhance and extend their servant leadership potential [17]. Accordingly, the purpose of the present case study is to explore how a community-based service-learning project implemented as part of the curriculum of a college course influenced potential future business leaders’ (i.e., undergraduate students) SL development.

The specifics of the service-learning project succeed a brief review of the relevant literature and theory. Next, the qualitative results (i.e., the student reflections completed at the end of the project) are presented through the lens of a servant leadership framework [1]. A discussion of the qualitative findings, limitations, and directions for future research is then provided before concluding with ideas for future service projects focused on servant leadership development in aspirant business professionals.

### 1.1. Theoretical Foundations

The current study incorporates both experiential and service learning to explore SL development. Classroom learning was combined with active learning while also requiring participants to engage with the local community and produce reciprocal service outcomes. As a result, participants combined classroom learning, active learning, and service learning to gain a detailed understanding of key leadership concepts and their real-world applications.

Experiential Learning Theory (ELT) [18] informed the development and execution of the case study. This theory explains the benefits an individual (in this case, the study participant) may receive from participating in a community-based service-learning project. Part of the value of experiential learning is that individuals, such as students, obtain a broad knowledge base and intellectual skills as well as personal and social responsibility through the integrative learning process [19]. In fact, students who have participated in learning communities (e.g., senior culminating experiences, and service-learning projects) have perceived greater knowledge retention and personal development [20].

According to Dewey [18], experiential learning occurs through a cycle of experience, reflection, and application, where learners engage with real-world situations to derive meaning and understanding. In this study, ELT was operationalized by first designing a service-learning project that took students out of the classroom environment and placed them in a real-world mentoring role within a community-based setting. This immersion into a practical, hands-on environment allowed students to experience firsthand the challenges and rewards of working with at-risk youth, aligning with Dewey’s emphasis on learning through direct engagement with authentic tasks.

The second phase of operationalizing ELT involved structured reflective practices, which were integral to transforming the service experience into a meaningful learning opportunity. After completing their service hours, students were required to engage in reflective writing, prompted by specific questions designed to encourage deep consideration of their experiences. This reflective process is a crucial component of ELT, as it facilitates personal growth and cognitive change by prompting students to analyze and internalize the lessons learned from their experiences. By connecting these reflections to broader leadership concepts, students can synthesize their practical experiences with theoretical knowledge, thereby enhancing their intellectual skills and fostering a sense of social responsibility. This deliberate alignment of experience, reflection, and application in the study mirrors the cyclical nature of ELT, making it a foundational framework for understanding how servant leadership attributes can be cultivated through service learning.

### 1.2. Servant Leadership Review

#### Conceptualizing Servant Leadership

The concept of SL is ancient, with roots stretching back thousands of years. An early reference comes from the Tao Te Ching, written around 500 BC in China, where Lao-Tzu asserts that the highest type of ruler is one of whose existence the people are barely aware [21]. This ancient philosophy introduced the notion that true leaders serve others selflessly, without seeking recognition. In the modern era, Robert K. Greenleaf, a former AT&T executive, is credited with popularizing the concept of SL in the business world. In his influential essay, “The Servant as Leader”, published in 1970, Greenleaf coined the term “servant leader” and went on to describe this leadership style as one that prioritizes the personal growth and well-being of others, emphasizing ethical conduct and the importance of doing good [22]. Greenleaf’s description of a SL is simple yet profound: “The servant leader is servant first… It begins with the natural feeling that one wants to serve, to serve first. Then conscious choice brings one to aspire to lead… The best test, and difficult to administer is this: Do those served grow as persons?” [4] (p. 27).

Since the emergence of Greenleaf’s ideas in the 1970s, various academic models of SL have been proposed. These models have adapted and expanded the original concept, adding unique perspectives and attributes. Notable contributions include works by Barbuto and Wheeler [7], Ehrhart [6], Laub [5], Liden, Wayne, Zhao, and Henderson [8], Sendjaya, Sarros, and Santora [9], Van Dierendonck [11], and Sun [12]. Each model has contributed a new dimension to the definition of SL, making the concept more robust and multifaceted (Table 1).

To illustrate the evolution and diversity of SL models, a timeline can be seen in Table 1. The broad range of attributes associated with SL, from empathy and healing to stewardship and community building, has led to numerous efforts to integrate these diverse perspectives. Such efforts have resulted in the model on which this study is focused. The focus of this study is a model created specifically for the purpose of developing undergraduate students’ SL abilities [1]. The three-sphere model revolves around a core motivation of altruistic calling which may reveal itself in leaders as the perception that one’s vocation, or mission in life, is to serve others. In the second sphere of the model are the central attributes of SL. These are constant over time and across contexts and include genuine caring, humility, and empathy (see Table 2 for definitions). The model concludes with a third sphere. In this sphere, the necessary leadership qualities beyond the core and central attributes may be utilized based on the population of followers, context, and situational variables. For example, leadership approaches such as establishing clear structures or asserting authority, may be essential when rebuilding an organization. However, these methods may not come naturally to someone who is inherently a servant leader.

In summary, SL is an enduring concept that has evolved significantly over millennia. From its ancient roots in the Tao Te Ching to its modern-day interpretations and models, SL emphasizes selfless service, ethical conduct, and the growth and well-being of others. The continuous development and adaptation of this leadership philosophy highlights its relevance and potential for fostering positive organizational and societal change.

### 1.3. Benefits of Servant Leadership

Numerous positive outcomes within a business environment have been linked to SL. This form of leadership can foster an open and trusting environment [26,27,28] as well as a culture of innovation [29]. Additionally, at the individual and team level, SL enhances organizational citizenship behavior [6,30], encourages collaboration between team members [31,32,33], and increases team and leader effectiveness and performance [31,34,35,36,37,38]. Servant leadership may also positively influence employee job satisfaction of employees [34,37] and decrease turnover intentions [39].

### 1.4. Servant Leadership in Sport

Regarding the significance of SL in sports, where the emphasis on winning can often overshadow the development of student athletes, consider the philosophy of the National Collegiate Athletic Association (NCAA). This nonprofit organization is responsible for regulating college athletes across the United States and emphasizes the importance of prioritizing the holistic development of student athletes. Its core purpose is “to govern competition in a fair, safe, and sportsmanlike manner, and to integrate intercollegiate athletics into higher education so that the educational experience of the student athlete is paramount” [40] (p. 3). Several of the NCAA’s core values include integrity, sportsmanship, respect, and fostering an inclusive culture [40]. Given this mission to serve student athletes, scholars have argued that SL could be the optimal model to support the NCAA’s mission and address the ethical issues in intercollegiate athletics in the United States [41]. Servant leadership emphasizes serving others, prioritizing the well-being and growth of individuals, and leading with a moral compass. This leadership style aligns with the NCAA’s values and its aim to enhance the educational experience of student athletes.

Further, in a review on leadership in sports, Peachey, Zhou, Damon, and Burton highlighted that the most frequently cited leadership article in sports management literature advocates for SL in intercollegiate athletics [42]. However, the gap between the advocacy for SL and its actual implementation in educational content suggests a need for more emphasis on this leadership style in sports management curricula, empowering educators to better prepare students to lead with integrity, prioritize the well-being of athletes, and create inclusive and respectful sport environments. Servant leadership could serve as a powerful tool to address ethical challenges in intercollegiate athletics, ensuring that the focus remains on the holistic development and education of student athletes. As the NCAA continues to strive for fair and safe competition while upholding its core values, embracing servant leadership could help fulfill its mission more effectively and foster a more ethical and supportive environment in collegiate sports.

The scholarship on SL in sports shows promise even though it is still a relatively new area of leadership research within the sports management discipline. In the realm of interscholastic sport, for example, servant-leader coaches were shown to produce players who were more satisfied, had higher intrinsic motivation, were more task-oriented, and demonstrated more self-confidence than non-servant-leaders [10,43]. Servant leadership has also been shown to enhance coach–athlete relationships, specifically contributing to the individual growth and moral development of athletes [44], as well as improving ethical climate and trust in followers [45]. This style of leadership has also been shown to enhance athlete satisfaction [46], positively influence student athletes’ immersion in the sport and athletic achievement [47] and improve coaching success [48]. Further, SL by interscholastic athletic directors has been revealed as an effective style by followers (head coaches) and resulted in head coaches who were more committed and had greater job satisfaction [49].

Recommendations have been put forth for fostering the growth of SL, advocating for students to participate in volunteer activities that entail direct engagement with the individuals they serve, with a preference for multiple visits, as suggested by Robinson, Neubert, and Miller [1]. These recommendations for experiential learning not only guide the service experience but also provide a framework for analyzing participant reflections, as outlined in the three-sphere model of SL [1]. Rooted in an altruistic calling, the SL model emphasizes the qualities of genuine caring, humility, and empathy. Definitions of core and central attributes of servant leadership according to the SL model are detailed in Table 2. This project functions as a follow-up and quasi-test of this service-learning approach for nurturing the development of servant leaders. The study is anchored in the SL framework, aiming to assess whether, in this context, service learning enhances student experiences regarding servant leadership.

## 2. Materials and Methods

### 2.1. Service-Learning Project Description

The service opportunity was coordinated by the instructor with the help of a service-learning coordinator office housed on campus. The Institutional Review Board (IRB) approved the study proposal, determining that it posed no potential harm to participants. IRB deemed that no identifying information would be used, and that no person would know whose statements might be used in the article, except for the student who wrote the reflection. Furthermore, because the study analysis occurred after students had concluded their college education and graduated, the IRB granted a waiver of informed consent and authorized the utilization of the reflections utilized for this study.

This case study included nineteen upper-level college students enrolled in a sports management course at a liberal arts university in the Southwestern United States. College students participated by serving as mentors for at-risk elementary school students (i.e., students who have higher a probability of failing a class or dropping out of school due to socio-economic concerns or domestic challenges such as parental absenteeism) during a local community center after-school program aimed at helping these students with homework and after-school recreation. As part of the class but before the service project began, students were presented with information on the importance of mentoring and guidelines for how to serve as an effective mentor by developing a partnership between someone with experience and someone who wants to learn. Servant leadership was not explicitly integrated into the course curriculum, ensuring that student reflections remained impartial and did not lean towards discussing SL attributes.

Students volunteered, forming groups of 3–4, at the community center for two hours a day, one day per week, over six weeks. This resulted in a total 12 h of service time. Mentors started each afternoon by helping students with homework assignments, lasting about an hour. Mentors then lead recreational activities outside such as kickball, basketball, running relays, and obstacle courses. Mentors and students also used the post-homework time for one-on-one conversations and relationship building. Student volunteers were required to keep a daily log of their activities and complete a final reflection evaluating the role and impact of the service-learning project on their personal growth and development. Participating mentors were also instructed to complete a final reflection of 300–400 words. For the final student reflections, students were prompted with the following questions: “Focus on how this service project impacted you. What have you learned from the experience?”; “What were you able to give, and what benefit did you receive in giving your time to help students?”; “What did you learn from the experience?”; “How has this impacted your perspective?”; “How will the experience impact your future?”. Completed final reflections were submitted online via Canvas, the institution’s academic online learning platform, and later collected for analysis.

### 2.2. Analysis

Content analysis serves as a method to dissect textual data and unveil underlying themes. Unlike thematic analysis, content analysis follows a top-down approach where themes are derived beforehand, often guided by theoretical or analytical interests [50]. Thematic analysis, in comparison, would have required the researchers to analyze the data, code them, identify them, review the themes in connection to the data, and then label the themes accordingly. Given that the current case study explores the interplay between service learning and the cultivation of SL, themes were predetermined based on an SL model that encompasses key characteristics of servant leaders like altruistic calling, genuine caring, humility, and empathy [1]. This approach aligns more with content analysis than thematic analysis. Definitions of SL attributes utilized for analysis are provided in Table 2.

Following the submission of reflections by mentors, the data were organized into an Excel spreadsheet, with any identifiable information removed to ensure participant anonymity. The researchers, working separately from each other, systematically categorized statements into the pre-established themes. Virtual meetings were then convened amongst the researchers to compare findings and address any discrepancies in coding and research agreement so as to avoid concerns about interrater reliability. This comparative process aids in identifying plausible themes and assessing statement alignment with each theme [51]. The comparative process was replicated three times over a two-month timeframe to facilitate both immersion and detachment. Each meeting lasted approximately 45 min. Immersion in the data is crucial for gaining a comprehensive understanding, while distancing enables researchers to evaluate the analysis from an objective standpoint [52,53]. During the first meeting, altruistic calling and genuine caring were coded with different color highlights. In the second meeting, humility and empathy were classified. Finally, during the third meeting, complete consensus was reached among researchers regarding statements that aligned with the established themes, and which statements were selected to be included in Table 2.

## 3. Results

Results were gathered from 19 upper-level (junior and senior) sports management students enrolled in a sports management course who also participated as mentors in an after-school program for at-risk elementary school youth. The mentors included 13 males and 6 females. Participants identified as White (11), Hispanic/Latino (7), and Black (1). Data were grouped into four themes: Altruistic Calling, Genuine Caring, Humility, and Empathy. Categorical definitions, summative results, and select statements are presented in Table 2.

Evident in the results is that service learning may have a positive impact on SL development. Participant statements present in the reflections were determined to fit within each of the pre-determined themes. While not every reflection touched upon all available themes, each reflection contained information that addressed at least one SL theme.

### 3.1. Altruistic Calling

Statements associated with the theme of altruistic calling showed up in 16 of the 19 reflections (84%). Expressions fitting into this category centered on students’ aspirations to sustain their service in diverse roles as students and in their forthcoming careers and personal lives. Moreover, comments highlighting the gratification and fulfillment of participants derived from the activity were prevalent. They felt they had positively influenced the students they mentored, which reinforced their sense of making a meaningful impact. The abundance of statements associated with serving again in the future suggests that students not only enjoyed the experience but that they felt like they were able to make a difference for someone less fortunate. This altruistic calling lies at the core of being an SL [1], and this project may have helped to awaken and develop this sense of altruistic calling in participants. At a minimum, participating in this service-learning project created an awareness of a previously unrealized desire present in participants. For example, in Statement 2, the participant mentions not only a desire to serve again, but that it was an experience that the participant did not know was needed. The idea that this project increased a desire to serve again is integral to developing the core motivation behind SL.

### 3.2. Empathy

The next most popular theme after altruistic calling was empathy. It was revealed in 15 of 19 statements (79%). Selected statements contributing to this theme centered on the social bonds forged between mentors and participants of after-school programs encompassing an intellectual grasp or empathetic experience of the feelings of after-school participants, a connection with the student life situations within after-school programs, the cultivation of patience through understanding, reciprocal learning, and the formation of connections between mentors and mentees based on shared learning disabilities.

The direct contact between mentors and mentees over multiple meetings in this case allowed for relationships to be forged and the mentors were able to vicariously experience the emotional states of followers. As these emotional states were communicated over time they became more thoroughly understood. Consider Statement 5. This statement refers to a situation where the mentor and mentee were able to connect because they both had been diagnosed with dyslexia, a learning disability characterized by difficulties with accurate and/or fluent word recognition and poor spelling and decoding abilities.

Additionally, numerous participants indicated that working with these children helped them recapture the experience of childhood and identify with the students (see Statements 1, 2 and 10). The mentors also noted they learned from their students (see Statements 2, 4, 6, 7 and 9). The ability of a leader to intellectually identify with followers and understand their psychological perspective is crucial to becoming a servant leader, as servant leaders must first understand the perspective of followers to better meet their needs and help them grow. Thus, the current results reveal that through the service-learning interactions, participants experienced growth in this key facet of SL.

### 3.3. Genuine Caring and Humility

Though genuine caring and humility appeared in lower frequency than other themes (9 of 19, 47%), these characteristics still manifested in nearly half of the mentors. As shown in Table 1, this service-learning project nurtured a genuinely caring attitude in mentors toward followers. Statements that focused on the reciprocal caring relationships developed between mentors and after-school program students were included in this theme. Development in this area helps aspirant leaders prioritize followers’ needs first, which is a core component of SL.

As well, many participants mentioned humility specifically (Statements 1 and 5), and others mentioned an increased understanding of the positive situations they may have taken for granted when they were children and before participating in this project (Statements 3 and 6). These statements remarked on the mentor’s evolving perspective, heightened appreciation for their own circumstances and opportunities, and gratitude for a childhood abundant in opportunities that might have been overlooked previously. An increased awareness of these qualities helps further develop SL in participants by encouraging reflection and fostering a deeper understanding of the emotions and thought processes of others. This heightened awareness can enhance leaders’ knowledge of their followers’ needs allowing them to take a proper course of action to meet those needs.

Finally, though not a predetermined theme, students expressed enjoyment with the service-learning project and were unanimous in stating how the project positively impacted their development and the perceived development of the students they mentored. Thus, the service-learning project was not only impactful for the students’ development of SL, but it also impacted their motivation to engage in prosocial, community-centered service efforts in the future. This demonstrates that the principles of SL, although often focused on the workplace, can extend beyond professional settings. The project fostered a sense of responsibility and care for the broader community, encouraging behaviors that may not necessarily involve colleagues or subordinates. As a result, students not only grew as leaders within the context of their careers but also as compassionate and proactive members of society, embodying the essence of SL in diverse aspects of their lives. This dual impact underscores the versatility of SL, suggesting that cultivating these qualities can lead to widespread benefits, both professionally and personally.

## 4. Discussion

In this study, a community-based service-learning project was incorporated into the curriculum of a college course to determine whether it would influence SL development in aspirant sports business professionals. The experience resulted in the participants showing growth in core facets of SL like altruistic calling, genuine caring, humility, and empathy. The participants also reported enjoyment in the service-learning project itself and an intent to be prosocial toward their community in the future.

Several reviews on SL leadership in sports (i.e., [1,42]) have advocated for the positive benefits of SL to intercollegiate athletics and the professional development of future sports professionals. For example, Robinson et al. [1] called for greater attention regarding how exactly SL can be incorporated into sports management curriculum and for evidence that it benefits the development of leadership skills in students. The current results support such calls by demonstrating how, in a case study scenario, service-learning opportunities can facilitate SL development in future business professionals. Moreover, the results support Dewey’s [18] beliefs on experiential learning as well as the operationalization of ELT to a sports management classroom, having demonstrated that student learners can indeed engage with real-world situations to derive meaning and understanding about a phenomenon. In this case, the phenomenon being SL.

The results of this study also support previous SL research even if the research was focused on athletes and coaches more so than sports management students. In the past, researchers have shown SL improves athlete growth and moral development [44], athlete satisfaction [46], and coach job satisfaction [49]. The students partaking in the SL-centered service-learning opportunity experienced similar outcomes. From their qualitative responses, it was clear they grew and experienced moral development, as evidenced by the participants indicating they would like to remain engaged with their local communities in the future. The participants were also satisfied with their experience and how the social exchanges and mentoring roles helped them develop SL skills. Thus, though not performed in an athletic context, the SL outcomes are similar and can be used to increase the generalizability of SL research to populations other than college athletes and coaches.

### 4.1. Study Limitations

This case study provides evidence that service learning may be a fruitful practice for developing SL, but the study is not without limitations. Though this is a case study, it would be beneficial to include a larger population in future studies. As well, this study relied on self-reported data. Future studies might consider gathering information from students, or an observational approach by including an observer to take notes on mentor-student interactions to include in the analysis. Further, it would be interesting to utilize a control group for comparison.

For the purposes of analyzing a proposed teaching method, themes for this study were determined a priori as suggested in prior research [1]. Though this strategy served its purpose, additional, different themes may have been protracted with a thematic analysis based on an inductive approach. It is possible that themes contained in the dataset may have been overlooked due to the pre-determined lens through which the data were viewed. The current case study results are promising, which should encourage future research in this area that includes a thematic analysis approach. Such analysis may reveal other important implications for developing future leaders.

An additional point to consider is that the themes analyzed in this study overlap somewhat, making categorization difficult. For example, discerning between empathy and genuine caring proved challenging, especially when considering statements that highlighted the bond formed between mentor and student. Upon completion of the analysis, the researchers categorized statements associated with emotional understanding as empathy. Statements categorized under genuine caring focused on wishing the best for followers. Through the review and process of immersing and distancing, researchers were able to reach a consensus on where terms should be classified, alleviating some of this concern. After several meetings, the researchers agreed on the final best fit for where to include statements. Even if some possible overlapping of statements and different themes exist, the selected statements in this case study still fall within the SL framework. In the future, researchers could provide definitions of themes to participants and ask more specific questions regarding each of the themes considered in this study. Following this process might more specifically target the development of participants in each of these areas and alleviate potential overlap.

Lastly, though informative, the three-sphere model does not explicitly detail how SL may interact with other leadership styles, such as transformational or ethical leadership, to influence follower (subordinate) reactions and behaviors. Thus, in the future, alternative theoretical approaches may prove useful to understanding SL development. For example, implicit leadership theory (ILT) considers follower perceptions and responses to leader behaviors. Followers form ILT of what leaders should (or should not) be and how they should act in a particular environment. Alignment between follower ILT and actual leader behavior results in better relationship formation and constructive follower behaviors [54]. Future scholarship should consider how SL may, either alone or in tandem with other leadership styles, influence subordinate reactions to leadership. For instance, if followers have ILT that includes self-regard and career advancement, then an approach to leadership that elevates followers’ needs like servant leadership may align well with follower leadership expectations and result in positive follower reactions.

### 4.2. Directions for Future Research

The present case study included sports management students completing a management course. In the future, investigations of the relationship between service learning and leadership development are needed across various populations and disciplines. Additionally, the question remains as to whether participants become more like SL in the future. That is, do participants really volunteer more as suggested by their reflections? The journals capture the moment, but evidence is needed as to whether the statements shared in the reflections become physical actions associated with the inward reality expressed during the service-learning experience. Follow-up also needs to be carried out to see if the participants, noted as aspirant business professionals, become more like SL when placed in leadership roles. Indeed, what becomes participants’ leadership styles as they move into the professional world? Longitudinal studies that follow participants into their professional careers, even if it is a small number of students, could help address these questions.

Along with the possibility of in-depth, longitudinal research, researchers might consider the development of an SL growth scale. An instrument such as this, one with items ranked on a Likert Scale, could include statements about how student experiences during a service project connect to facets of SL such as altruistic calling, genuine caring, humility, and empathy. An example item for altruistic calling might read, “After participating in this service activity, I have become more aware of my desire to serve in the future”. Though this instrument would not address the long-term implications of service-learning experiences and the development of SL, it would complement the qualitative results found in reflection journals and give researchers a tool to demonstrate a correlation between specific service activities and SL.

Finally, future studies should consider what those being served (e.g., the at-risk youth being mentored) gain from the experience. One of the best tests of SL effectiveness is to determine if followers are more likely to become servant leaders themselves [4]. Numerous questions about those being served are ripe for exploration. What do mentees get out of the service-learning project? How is their development impacted? Do they perform better in school? What is the connection between their school performance and the mentor–mentee interactions? Are they more likely to become servant leaders themselves because of their mentor–mentee relationship? Addressing questions about mentee perceptions of their mentor, which could align with ILT, and how this relationship is perceived to have influenced mentee school performance, life satisfaction, and development as a leader would make for an interesting avenue of future exploration.

### 4.3. Suggestions for Service-Learning Projects

Service-learning experiences represent a relatively simple and cost-effective way for students to develop SL and connect with their local communities. The current case provides a service-learning example that connected student mentors with at-risk elementary school youth. Another service-learning project that may be beneficial to students is to have them host a Special Olympics Unified Field Day. The Special Olympics Unified Sports website describes unified sports as an inclusion initiative focused on bringing together those with and without disabilities to participate on the same team, aimed at fostering friendships through the development of understanding and empathy [55]. Students in a sports management setting could arrange and oversee an event. Requirements of the learning experience might include analyzing ADA compliance requirements, formulating emergency preparedness plans, gathering volunteers, and organizing specific activities. Ultimately, any activity where the service would meet the needs of those being served while bringing students into direct contact with them could serve as a quality service-learning opportunity.

Students involved in this case study were also not directly informed about SL prior to or after the service project. Alternatives to consider are teaching about SL beforehand or teaching about SL after the service project concludes. The former approach would allow students to place an emphasis on developing SL attributes in themselves as they serve and lead. Students could then complete a reflection based on the SL model. The latter approach would have participants learn about SL and then analyze their reflections for SL themes, thus connecting the practice of serving with reflection and analysis based on an informed understanding of SL attributes.

## 5. Implications for Servant Leadership Development

The findings from this study highlight the significant role that community-based service-learning projects can play in the development of SL qualities among aspiring sports management professionals. By actively engaging students in meaningful service activities that prioritize the needs of others, the study underscores how experiential learning can facilitate the growth of core SL attributes such as altruistic calling, empathy, genuine caring, and humility. These attributes are critical for future leaders who aim to foster ethical, inclusive, and supportive environments in their professional lives. The reflective component of the service-learning experience further reinforces the internalization of these values, encouraging students to consider the broader social impact of their leadership actions. This approach not only nurtures the personal and professional growth of students but also aligns with the broader objectives of sports management education, which seeks to prepare individuals to lead with integrity and a commitment to the well-being of others.

Furthermore, the study’s implications extend beyond the classroom, suggesting that incorporating structured service-learning projects into leadership development programs could be a powerful tool for cultivating SL across various disciplines. The research indicates that when students are given opportunities to get out of the classroom environment and engage with and reflect on real-world challenges, they are more likely to develop a leadership style that is both empathetic and ethically grounded. Institutions and organizations aiming to develop future leaders should consider integrating service learning as a key component of their training programs, as it provides a practical framework for students to practice and refine SL skills in a controlled yet impactful setting. By doing so, these programs can contribute to the creation of a new generation of leaders who are not only capable of making strategic decisions but also committed to serving and uplifting their communities.

## 6. Conclusions

The aim of this study was to investigate a proposed teaching strategy focused on developing SL via community-based service learning [1]. From the analysis of the reflection journals, it appears that participating in a service-learning project may be an influential practice for developing SL. Following the completion of their roles as mentors and subsequent completion of written reflections, the findings underscore the efficacy of service learning as a robust approach for instructing students about servant leadership as well as cultivating SL attributes within themselves. Instructors hoping to advance SL development in their students may want to consider including this process of service learning and reflection in future course planning. For research, one avenue scholars might consider is the development of an SL growth scale that could empirically measure whether students perceive their development in aspects of SL such as genuine caring and humility. Through deductive scale development [56], which has been performed extensively with scale development in sports management research, scholars could create a measurement tool that would complement in-depth qualitative exploration into the facilitation of servant leaders in sports.

## Figures and Tables

**Table 1:** Timeline of Common Servant Leadership Models.

Year	Author(s)	Model Description	Key Attributes
1970	Greenleaf [22]	The Servant as Leader	Serving first, personal growth of others, moral high ground, ethical leadership
1999	Laub [5]	Organizational Leadership Assessment	Valuing people, developing people, building community, displaying authenticity, providing leadership, sharing leadership
2004	Ehrhart [6]	Servant Leadership Behavior Scale	Emotional healing, creating value for the community, conceptual skills, empowering, helping subordinates grow and succeed, putting subordinates first
2006	Barbuto and Wheeler [7]	Dimensions of Servant Leadership	Altruistic calling, emotional healing, wisdom, persuasive mapping, organizational stewardship
2008	Sendjaya et al. [9]	Servant Leadership Behavior Scale	Voluntary subordination, authentic self, covenantal relationship, responsible morality, transcendental spirituality, transforming influence
2008	Liden et al. [8]	Servant Leadership Questionnaire	Conceptual skills, empowering, helping subordinates grow and succeed, putting subordinates first, behaving ethically, emotional healing, creating value for the community
2011	Van Dierendonck [11]	Servant Leadership Survey	Empowerment, humility, standing back, authenticity, forgiveness, courage, accountability, stewardship
2013	Sun [12]	Integrated Servant Leadership Model	Calling to serve, agape love, humility, empathy, integrity, trust, vision, empowerment, service orientation
2018	Robinson, Neubert and Miller [1]	3 Sphere Model and Teaching Recommendations	Altruistic calling, Genuine Caring, Humility, Empathy Service Learning and Reflection

**Table 2:** Categorical Definitions, Results, and Select Statements.

Attributes and Results	Definition and Statements
**Altruistic Calling** (16 of 19, 84%)	one’s deeply held beliefs that create a strong urge toward a particular way of life. When calling is altruistic in nature, a person is then motivated to promote the well-being of others, even at risk or cost to one’s self.
Statement 1	“In the future I wish I could spend more time with a group like that and even mentor maybe just one kid and watch them grow up and learn with your help. I would also like to serve again next year because I know how helpful and how much it means to the kids that I was there helping”.
Statement 2	“Overall my experience at Doyle was amazing I would definitely do this again. This experience was something I didn’t know I needed”.
Statement 3	“I hope to continually make efforts to volunteer and go and help the kids in any way that I can”.
Statement 4	“I definitely want to try and volunteer here again next year… Overall, it was a very rewarding experience for me and I’m very glad I was able to participate”.
Statement 5	“This will sound selfish, but I was able to give them my time. I am a full-time college student athlete who also has two jobs and is involved in other organizations. For me to say I was excited to add another thing on my to do list for Mondays would be a lie. However, the Doyle Center immediately became the most important to me”.
Statement 6	“I haven’t done too much volunteering, but after this I might volunteer more to see if I can have an impact on others like the kids we volunteered with”.
Statement 7	“Holding this with me to my future goals even furthers my dream of giving back to my hometown and developing areas. I’ll definitely be trying to go back to see the kids and again and volunteering as much as I can!”
**Genuine Caring** (9 of 19, 47%)	Based on a selfless, unconditional love known as agape love [23]; not merely behavior, but an expression of one’s true inner attributes [24].
Statement 1	“I was a little timid on my first visit not really knowing what to expect from the program, as well as, the kids in it. However, once they arrived from school, I jumped right in to help and created a bond with several of them within even the first few minutes. I enjoyed my time working with these kids and hope that they will all prosper in the rest of their lives”.
Statement 2	“I see them as siblings and I just want the best for them”.
Statement 3	“It allowed me to see how much of an impact I can make just by showing them that I care about their success in their life and that feeling is something that can’t be matched”.
Statement 4	“They have someone to help them with homework and to ask them about their day, someone who cares about them”.
Statement 5	“Saying goodbye to them was the hardest thing. Because I grew attached to them. I just wanted to keep seeing them grow and become better students and people”.
Statement 6	“Nothing is better then having one of the kids run up to you and give you a hand made thank you card that they made in art class that day; it means the world to me and shows that even though I was there for a class assignment I had a major impact on theses kids lives in just 6 visits or fewer”.
**Humility** (9 of 19, 47%)	Having a modest opinion or estimate of one’s own importance; keeping a realistic perspective on one’s position and capabilities; involves prioritizing the needs of others above one’s own [25].
Statement 1	“Getting to meet the kids and understand the way they are growing up and living life everyday was honestly a very humbling experience for me as well”.
Statement 2	“I like to think I am grateful, but after spending time with the kids I learned quickly that I am not”.
Statement 3	“I feel like I was very lucky as a child growing up because most of the kids didn’t have anyone but us. It was very sad to see that. I feel like I learned that don’t take your life for granted because there is always another kid or adult that wishes they can be in you place”.
Statement 4	“I learned to never take what I have for granted and to never be selfish in life because life is literally what you make it”.
Statement 5	“This experience very much humbled me”.
Statement 6	“Though it does show to me just how much I might have taken that for granted in my own life, it is hard to picture how much more I had going for myself than these kids do for them. It really shows how much in my early life I took for granted”.
**Empathy** (15 of 19, 79%)	Intellectual identification with or vicarious experiencing of the feelings, thoughts, or attitudes of another; ability to be aware of the emotional states of others without those states being explicitly communicated [12]; allows leaders to understand the point of view and psychological perspective of others [7].
Statement 1	“One of the biggest things that impacted me is seeing myself in these kids and it is an experience I will never forget”.
Statement 2	“They all taught me things Gee taught me to sympathize and reason with people, Sophia taught me life can be fun you just have to make it fun, Joseline taught me to smile, Danny and his sister taught me family is important, and Yasmine taught me freedom. They taught me more about myself than I could have ever expected. In life I was very strict and up tight about things good or bad I just wanted things to be perfect everyday all day. Working with them allowed me to remember what it was like to be a kid and not have things to worry about, even if it was just a few hours”.
Statement 3	“I wonder if these kids get the attention they need at home and I feel like they really enjoyed being at Doyle. Especially being from a low income area, I wonder if they have father figures or an older brother or an uncle who cares for them”.
Statement 4	“Even though we are supposed to be teaching them things, I learned so much from them. The biggest thing they taught me without even knowing it was patience... At first, I would get somewhat frustrated, but I quickly learned how to be patient with them because they may not have a structured home life where their parents sit down and teach them”.
Statement 5	“The kid I worked with the most was Sophia, she really seemed to like me, and I was able to help her learn because she has dyslexia and so do I, so I was able to teach her some tricks to learn and read more efficiently”.
Statement 6	“I have also noticed how these students look up to us, we are role models for them, and also how I could relate to their lives. I grew up in single parent home just like some of the children. I wanted just like them to have older people to help me with homework or hangout. It was an awesome time getting to know these students. I know that Akaziah loves to read about and play soccer. Her brother Dani loves to be outside and play tag, in which we played almost every day we were there. Gerrado loves wolves and playing basketball. His sister Joclyn is great with multiplication and wants to be a veterinarian when she gets older”.
Statement 7	“While there I did make a bond with a few of the students and they became the kids that asked me to help them regularly with their homework. Their names were Sophia and Joseline and they were sisters. Joseline was the older sister and Sophia was the younger sister. One interesting thing that I learned about Sophia is that she learned in both Spanish and English. Some of her reading material would be in Spanish and she would have to translate it to English. Which for me was kind of hard because I did not know a lot of Spanish so, Sophia was teaching me something”.
Statement 8	“Coming from a community where rural, underprivileged kids are prevalent throughout, this allowed me to immerse myself into their lives and a “behind the scenes” look on what they go through on a daily basis and what they have to deal with at school and at home”.
Statement 9	“Some things that I personally have learned from the experience is that you never know what someone else is going through until they tell you themselves. I found that regardless of where you come from or where you are, kids are just the same wherever you go. They are all just waiting for a person to look up to and learn from. They all just want a place to have fun and to play with their peers”.
Statement 10	“Getting to play with kids was my favorite thing because it made me feel like I was a young kid again and brought back my imagination”.

## Data Availability

The data that support the findings of this study are available from the corresponding author upon reasonable request.
